# Prediction of drugs having opposite effects on disease genes in a directed network

**DOI:** 10.1186/s12918-015-0243-2

**Published:** 2016-01-11

**Authors:** Hasun Yu, Sungji Choo, Junseok Park, Jinmyung Jung, Yeeok Kang, Doheon Lee

**Affiliations:** Department of Bio and Brain Engineering, KAIST, 291 Daehak-ro, Yuseong-gu, Daejeon, 305-701 Republic of Korea; Bio–Synergy Research Center, 291 Daehak–ro, Yuseong–gu, Daejeon, 305-701 Republic of Korea

**Keywords:** Drug repositioning, Network biology, Directed network

## Abstract

**Background:**

Developing novel uses of approved drugs, called drug repositioning, can reduce costs and times in traditional drug development. Network-based approaches have presented promising results in this field. However, even though various types of interactions such as activation or inhibition exist in drug-target interactions and molecular pathways, most of previous network-based studies disregarded this information.

**Methods:**

We developed a novel computational method, Prediction of Drugs having Opposite effects on Disease genes (PDOD), for identifying drugs having opposite effects on altered states of disease genes. PDOD utilized drug-drug target interactions with ‘effect type’, an integrated directed molecular network with ‘effect type’ and ‘effect direction’, and disease genes with regulated states in disease patients. With this information, we proposed a scoring function to discover drugs likely to restore altered states of disease genes using the path from a drug to a disease through the drug-drug target interactions, shortest paths from drug targets to disease genes in molecular pathways, and disease gene-disease associations.

**Results:**

We collected drug-drug target interactions, molecular pathways, and disease genes with their regulated states in the diseases. PDOD is applied to 898 drugs with known drug-drug target interactions and nine diseases. We compared performance of PDOD for predicting known therapeutic drug-disease associations with the previous methods. PDOD outperformed other previous approaches which do not exploit directional information in molecular network. In addition, we provide a simple web service that researchers can submit genes of interest with their altered states and will obtain drugs seeming to have opposite effects on altered states of input genes at http://gto.kaist.ac.kr/pdod/index.php/main.

**Conclusions:**

Our results showed that ‘effect type’ and ‘effect direction’ information in the network based approaches can be utilized to identify drugs having opposite effects on diseases. Our study can offer a novel insight into the field of network-based drug repositioning.

**Electronic supplementary material:**

The online version of this article (doi:10.1186/s12918-015-0243-2) contains supplementary material, which is available to authorized users.

## Background

A drug is a chemical compound used to treat diseases and it usually has opposite effects to disease states. The traditional drug development remains expensive and time consuming process with low success rate. Developing a new drug to market takes about 15 years and 1 billion US dollars [1] and more than 85 % of drugs are failed to be released [2]. The identification of new therapeutic indications for existing drugs suggests an alternative for this situation [3]. The repurposing of approved drugs reduces costs and times involved in early phase of drug discovery.

*In silico* methods have provided candidates for novel uses of existing drugs. With the emerging technologies generating gene expression profiles, one of prevalent computational approaches to identify novel indications for existing drugs is based on drug and disease gene expression signatures from public databases [4–7]. These methods predicted drug and disease pairs showing therapeutic relationships through comparison of signatures of drugs effect and diseases pathophysiology. They discovered some previously uncharacterized uses of known drugs to diseases. However, these studies may have low precision since gene expression profiles, especially for drugs, can be generated under various conditions such as different tissue, cell line/types, and different doses of drugs [5].

**Fig. 1:**
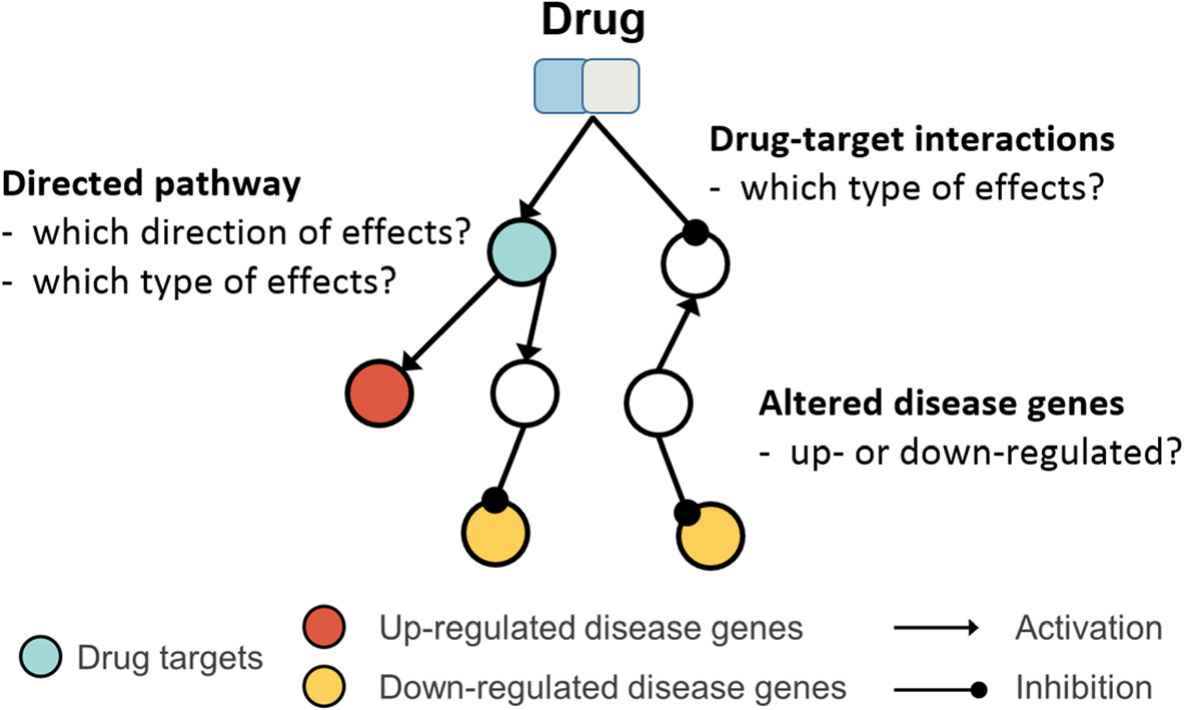
The way a drug has effects on disease genes. The entire path from a drug to disease genes consists of ‘effect type’ such as activation or inhibition, ‘effect direction’, and ‘altered states of disease genes’ like up- or down-regulated states in disease patients

Other recent popular *in silico* approaches in drug repositioning are network-based approaches including: 1. prediction of interactions between drugs and target proteins and using them for drug repositioning [8–11]; 2. prediction of drug effects to disease genes on biological networks [9, 12, 13]. These network-based studies have revealed promising results. However, even though there are diverse types of drug-drug target interactions such as activation or inhibition, the first type of network-based methods using drug-target interactions (DTI) regarded drug-target protein interactions as binary interactions, except for one [11], which did not illustrate how this method could be used in drug repositioning. The second type of network-based methods using molecular biological networks did not fully take into account the way drugs affect to disease genes because these methods did not consider ‘effect types’, ‘effect directions’, and ‘altered disease genes’ in diseases (Fig. 1). Thus, candidate drugs for the treatment of diseases from previous studies could activate over expressed genes in the disease state or inhibit under-expressed genes and could accelerate progression of the disease. In addition, candidate drugs from previous studies could not exert influence on diseases genes due to ‘effect direction’ (Fig. 1).

Here, we proposed a novel approach to identify novel indications of existing drugs by predicting drugs having opposite effects on altered disease genes. Our hypothesis is that if a drug compensates differentially regulated states of disease genes in patient, then that drug has the potential to be a therapy of the disease. Entire routes from drugs to disease genes are considered with the ‘effect types’, ‘effect directions’, and ‘altered disease genes’ information: interactions between drugs and drug targets; directed pathway between drug targets and disease genes; regulated states of disease genes in patients. This approach is termed as Predicting Drugs having Opposite effects Disease genes (PDOD).

**Fig. 2:**
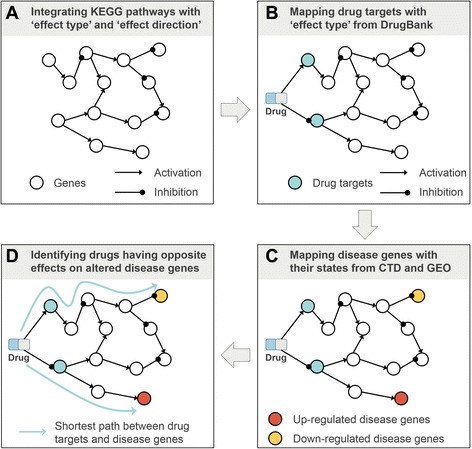
Overview of PDOD. **a** Integrating pathways whose edges have ‘effect type’ and ‘effect direction’ information. **b** Mapping drug-drug target interactions on an integrated pathway with their ‘effect type’ and ‘effect direction’. **c** Mapping disease genes with their regulated states in patients. **d** Predicting drugs having opposite effects on states of disease genes in disease states based on shortest paths

In our PDOD approach, we integrated directed pathways having ‘effect type’ and ‘effect direction’, mapped drug-drug target interactions with their ‘effect types’, and mapped disease genes with their differentially regulated states. Based on this entire path, we predicted a drug whose targets are close to disease genes and activate down-regulated disease genes or inhibit up-regulated disease genes in patients as a therapy for the diseases (Fig. 2 and Additional file 1: Figure S1). We also showed that our method would be able to verify known uses of drugs and predict novel indications for existing drugs.

## Methods

### Datasets

To identify how drugs influenced on disease genes, we considered the shortest paths from drugs to diseases. We collected directed molecular pathways, drug-target protein interactions, and disease related genes with their regulated states from various data sources. The datasets used in our method are described here.

#### Integrated directed pathways

We constructed a directed backbone network having ‘effect type’ and ‘effect direction’ by integrating Kyoto Encyclopedia of Genes and Genomes (KEGG) pathway [14]. Although KEGG pathway does not cover entire molecular entries and pathways in human, it provides manually curated pathways with highly reliable relations having ‘effect type’ and ‘effect direction’ information. We downloaded KEGG Markup Language (KGML) files of 286 human pathways using KEGGgraph package [15]. We only used gene entries and relations with obvious ‘effect direction’ like activation, inhibition, expression, and repression. Then, the 166 human pathways including signal transduction and transcription pathways were merged into a Directed KEGG pathways (DKEGG) consisting of 3,307 nodes and 28,808 directed edges. Each node in DKEGG is a gene and each edge represents a relation with ‘effect type’ and ‘effect direction’ from gene A to gene B, for example, gene A activates (inhibits) gene B. In DKEGG, if the effect type from gene A to gene B was ‘activation-like effect types’ like activation or expression, we assigned the edge weight from node A to B as ‘+1’ and if the effect type from node A to B was inhibition or repression, the edge weight from node A to B was set to ‘-1’.

#### Drug-drug target interactions

Drug-drug target interactions were obtained from DrugBank database (version 4.0) [16]. We collected drugs which possess known ‘activation-like’ or ‘inhibition-like’ drug-drug target interactions. We regarded activation, agonist, activator, simulator, and partial agonist interactions as ‘activation-like drug-drug target interactions’ and inhibition, inhibitor, antagonist, negative modulator, inverse agonist, suppressor, inhibitor (competitive), partial antagonist, reducer, and blocker as ‘inhibition-like drug-drug target interactions’. We filtered out drugs whose targets were not included in DKEGG. Finally, we extracted 2,434 drug-drug target interactions for 364 drug targets (genes) and 898 drugs.

#### Selection of diseases

We selected diseases satisfying several conditions: 1. Known disease-gene associations with direct evidence are in Comparative Toxicogenomics Database (CTD) [17]; 2. Diseases related tissues are clear; 3. Gene expression profiles of disease cases and controls obtained from related tissues exist in Gene Expression Omnibus (GEO) database [18]. Based on these conditions, we selected original 16 disease. Gene expression data information and related tissues about 16 diseases we firstly chose were shown in Additional file 2: Table S1.

#### Disease genes with their altered states

Disease-gene associations for 16 diseases were collected from CTD. We only used disease-gene associations having direct evidences. MeSH IDs [19] of diseases and the number of the associated disease genes are described in Additional file 2: Table S1.

We assigned altered states of disease genes using gene expression data. We downloaded microarray gene expression datasets for each disease from GEO. The altered states of disease genes were assigned to up-regulated or down-regulated if disease genes are significantly differentially expressed genes between disease and control samples. We identified significantly differentially expressed genes using linear models and empirical Bayes methods (limma) [20] representing several practical advantages compared to other methods [21]. We found altered disease genes of 16 diseases whose FDR corrected *p*-values were less than 0.05. Five diseases, Alzheimer’s disease, bipolar disorder, chronic obstructive pulmonary disease, idiopathic pulmonary fibrosis, and major depressive disorder, were excluded because none of their disease genes was significantly differentially expressed in disease states. Then, we checked whether differentially expressed disease genes exist in DKEGG or not. Multiple sclerosis was removed because its differentially expressed disease genes do not exist in DKEGG. Pulmonary hypertension was also filtered out because a differentially expressed disease gene is an isolated node in DKEGG. Finally, nine diseases were used in our further analysis.

#### Collecting therapeutic drug-disease associations

The chemical-disease association file was downloaded from CTD. MeSH IDs, presented in Additional file 2: Table S1, were utilized to match nine diseases in our study to diseases in the chemical-disease association field. We collected answer drugs for each disease which mean the drugs could be used to treat the diseases. The chemicals were employed as the answer sets in the further analysis according to two criteria: 1. Chemicals have therapeutic associations for diseases; 2. Chemicals have DrugBank ID and drug-drug target interactions with their effect types. The entire list of answer drugs with DrugBank ID for each disease is shown in Additional file 3: Table S2.

### Resolving conflicts in DKEGG

We hypothesized drugs whose targets have short distances to disease genes and activate (inhibit) down (up)-regulated genes in disease states can be used to treat the diseases. To calculate distances between drug targets and disease genes, we inferred all possible shortest paths from each drug target to each disease gene using Breadth-first-search algorithm in DKEGG with ‘effect type’ and ‘effect direction’. In our approach, a distance from gene A to gene B can be a negative value if gene A inhibits gene B.

In directed biological pathways, there exist conflicts. Conflict refers to a situation that two or more contradictory relations coexist in directed biological pathways [22]. Conflicts usually occur in biological pathways due to different biological contexts [23], for example, a dual way of TAK1 working corresponding to different cellular contexts [24]. In addition, conflicts were also observed in shortest paths from node A and node B in the directed pathway. For example, there were cases that gene A activated gene B in one shortest path while gene A inhibited gene B in other shortest paths: gene A activates gene C and gene C activates B; gene A activates gene D and gene D inhibits B.

To resolve this problem, we searched all possible shortest paths and set a distance *d*_*c*_(*r*, *g*) from a drug target *r* to a disease gene *g* with the consideration of the conflict problem by

1 $$ {d}_c\left(r,g\right)=\frac{n_a+{n}_i}{n_a-{n}_i}\left|d\left(r,g\right)\right| $$

where *n*_*a*_ is the number of ‘activation-like shortest paths’, *n*_*i*_ is the number of ‘inhibition-like shortest paths’, and |*d*(∙)| is an absolute value of distances of shortest paths between a drug target and a disease gene. Whether a shortest path is an activation or inhibition-like path is determined by the sum of the number of inhibition and repression edges. If the sum of the number of inhibition and repression edges is an even number including zero in the shortest path, it is regarded as an activation-like path and if an odd number, an inhibition-like path. The sign of *d*_*C*_ indicates whether the drug target would activate or inhibit the disease gene. When the number of activation-like shortest paths is greater than inhibition-like shortest paths, the sign of *d*_*C*_ is positive, or vice versa. The absolute value of *d*_*C*_ reveals how reliable the effect from a drug target to a disease gene is. As the number of activation and inhibition-like paths are similar, the absolute value of *d*_*C*_ increases than an original absolute value of distances |*d*(∙)| and it represents the drug target would give little influence on the disease gene. If the number of activation and inhibition-like paths are equal or a drug target *r* and disease gene *g* are not connected in DKEGG, *d*_*C*_(*r*, *g*) will be a value of infinity. The distribution of *d*_*C*_ between all genes in DKEGG is presented in Additional file 4: Figure S2.

### A score between a drug and a disease

For each drug and disease pair, we calculated a score using drug-target interactions, distances from drug targets disease genes with consideration of conflicts, and the regulated states of disease genes. In our PDOD approach, a PDOD score between a drug *R* having drug targets *r*_*i*_ and a disease *G* with its associated disease genes *g*_*j*_ was computed as below:

2 $$ \begin{array}{l}\\ {}\mathrm{PDOD}\kern0.5em \mathrm{score}\left(R,G\right)=\frac{1}{n_g{n}_r}{\displaystyle \sum_{i=1}^{n_r}}{\displaystyle \sum_{j=1}^{n_g}}sgn\left({r}_i,\ {g}_j\right)\times \frac{1}{1+{\left|\frac{d_c\left({r}_i,{g}_j\right)}{\alpha}\right|}^2}\\ {}\end{array} $$

3 $$ sgn\left({r}_i,\ {g}_j\right)=i\left({r}_i\right)\times sign\left({d}_c\left({r}_i,{g}_j\right)\right)\times s\left({g}_j\right) $$

where *α* is the parameter of a bell shaped function meaning the half width of the bell shaped function, *n*_*r*_ is the number of drug targets of the drug *R*, *n*_*g*_ is the number of disease genes of the disease *G*, *i*(*r*_*i*_) is the corresponding value for the predefined ‘effect type’ of drug-target interactions and it is assigned to ‘+1’ for ‘activation-like drug-drug target effect type’ and ‘-1’ for ‘inhibition-like drug-drug target effect type’, *s*(*g*_*j*_) is ‘+1’ in case that *g*_*j*_ is a down-regulated gene in disease states and ‘-1’ in case of an up-regulated-gene, and *sign*(∙) is the sign function. Thus, the function *sgn*(*r*_*i*_ , *g*_*j*_) has a positive value when a drug has on opposite effect on a disease gene *g*_*j*_ through a drug target *r*_*i*_. We set our scoring function as a bell shaped function since we thought that if the distances from drug targets to a disease genes were longer than the specific distance, *α*, the possibility that the drug had an effect on the disease genes would decrease rapidly. Thus, an absolute value of our PDOD score is in inverse proportion to the norm of *d*_*c*_(*r*_*i*_, *g*_*j*_). We divided our scores by the number of drug targets of the drug because we thought target proteins which did not affect disease genes might act as ‘off-targets’ for treatment of diseases and these targets could lead to unexpected effects such as side effects [25]. Our scores were also divided by the number of disease genes for normalization between diseases and thus our scores had values between −1 and 1. Finally, in our scoring function, a drug will receive a high score in case that drug targets of the drug are close to disease genes, drugs targets activate down-regulated disease genes and inhibit up-regulated disease genes in patients, and the number of off-targets is small.

## Results and discussions

We applied our PDOD approach to nine diseases, adenocarcinoma of lung, acute myelocytic leukemia, astrocytoma, asthma, glioblastoma, oligodendroglioma, Parkinson disease, schizophrenia, and thyroid carcinoma. For each disease, we estimated the possibility of 898 drugs for the treatment of each disease based on PDOD scores which use drug-drug target interactions with ‘effect type’ from DrugBank, shortest paths from drug targets to disease genes with the consideration of conflicts in DKEGG, and changed states of disease genes in the disease with disease genes from CTD and expression profiles of GEO. The distribution of PDOD scores between all drugs and nine diseases we used presented in in Additional file 4: Figure S2–S3.

### Performance of PDOD and previous methods

#### Comparison to a network-based method without directional information

To verify the importance of directional information used in PDOD, we make a Predicting Drugs having effects on Disease genes (PDD) score which uses drug-drug target interactions without ‘effect type’, paths from drug targets to disease genes on DKEGG without ‘effect type’ and ‘effect direction’, and disease genes without their regulated states.

**Fig. 3:**
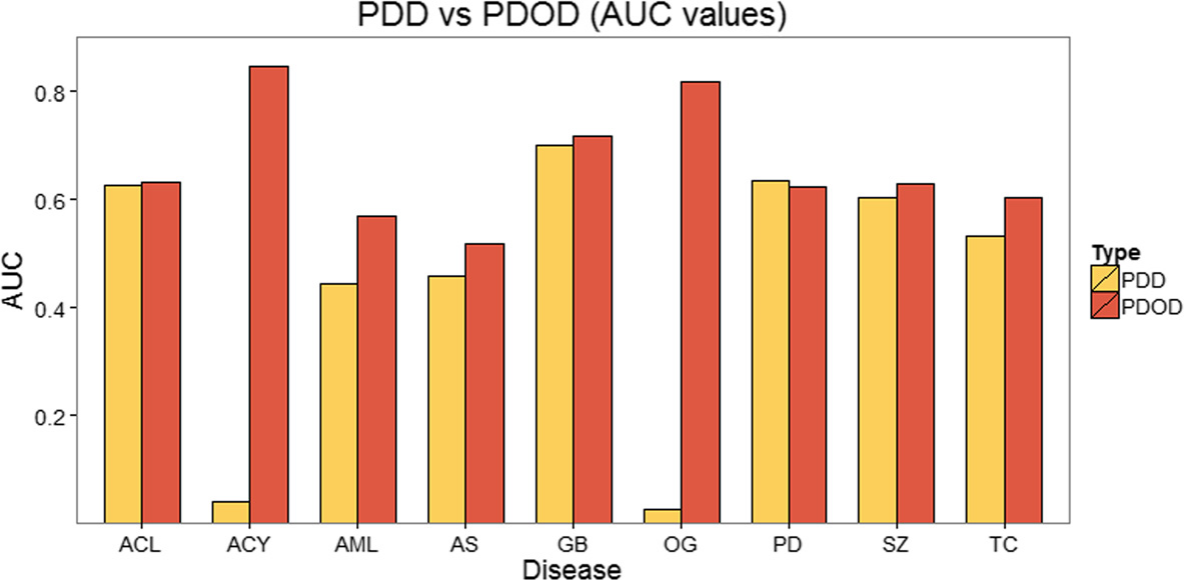
Comparison of PDOD with PDD. The AUC values of PDOD and PDD for nine diseases

We calculated PDOD scores and PDD scores between all 898 drugs and each disease respectively and compared performance of PDOD to PDD for predicting known drugs of each disease from CTD. Fig. 3 shows the area under receiver operating characteristic curves (AUC) values of PDOD and PDD approaches for nine diseases. As shown in Fig. 3, PDOD shows better performance than PDD in the prediction of answer drugs for diseases except for Parkinson disease. This illustrates the importance of directional information used in PDOD. We set a value of parameter *α* to three for AUC values of both PDOD and PDD in Fig. 3. AUC values through various *α* values are given in Additional file 5: Figure S4–S12. As shown in figures of Additional file 5: Figure S4–S12, some diseases like adenocarcinoma of lung and schizophrenia revealed slightly different AUC values according to values of parameter *α* and they have high AUC values when *α* is bigger than three. It may be related to results from the previous work [26] which shows that the averages of distances between drug targets and disease genes are dependent on the types of diseases.

#### Comparison to a previous method without directed network

Our approach was also compared with a previous method using gene expression profiles of both drugs and diseases [6, 7]. These previous approaches are analogous to PDOD in that they intended to identify drugs likely to have complementary effects on a disease state as therapies of the disease. Thus, we compared performance of PDOD, which is a network–based approach, to the previous method using only gene expression profiles for prediction of known therapeutic relationship between drugs and diseases.

**Fig. 4:**
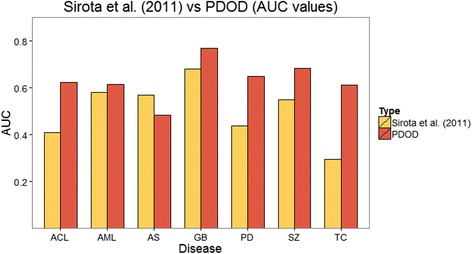
Comparison of PDOD with a previous method using only gene expression profiles. The AUC values of PDOD and a previous method which uses gene expression profiles of drugs and diseases

Disease gene expression signatures of the previous method were obtained identical datasets from GEO (Additional file 3: Table S1). We assigned that the numbers of up- and down-regulated disease gene expression signatures for each disease are equal to the numbers of up- and down-regulated disease genes used in PDOD. With disease gene expression signatures, the enrichment score (ES) introduced in Connectivity Map [6] was used for determining the potentiality of a drug for the treatment of the disease. A drug having high negative enrichment score was expected to repress the disease. Since some of answer drugs did not have drug exposure expression data in Connectivity Map and some drugs existing in Connectivity Map did not have drug-target interactions used in our approach. Thus, the intersection of two drug sets, 317 drugs, was used in this evaluation. In this process, the answer drug of astrocytoma and oligodendroglioma are filtered out. Thus, we compared performances of PDOD and the previous method for seven diseases (Fig. 4). Figure 4 indicates PDOD using directed network usually outperformed a previous approach using only gene expression signatures.

### Potentiality of high ranked drugs in PDOD as therapies

Top 10 ranked drugs receiving high scores in our PDOD for whole diseases are revealed in Table 1. All of the ten drugs getting high scores are not included in answer drugs for each disease. We investigated published literature which can support the possibility of uses of these drugs for the treatment of the disease. Six drugs have supporting evidence in literature for their expected usages [27–36]. These results show the potential of our PDOD approach for identification of novel drug indications.

**Table 1:** Top 10 ranked drugs in PDD and their potentiality for the therapy of diseases

Disease (MeSH ID)	Drug (DrugBank ID)	Description in DrugBank	Supporting evidence from literature
OG (D009837)	Tetracycline (DB00759)	Bacterial infections	[27]
TC (D013964)	Clomifene (DB00882)	Female infertility due to anovulation	n/a
TC (D013964)	Fulvestrant (DB00947)	Metastatic breast cancer	[28]
TC (D013964)	Ospemifene (DB04938)	Dyspareunia	n/a
TC (D013964)	Cetuximab (DB00002)	Metastatic colorectal cancer	[29, 30]
TC (D013964)	Gefitinib (DB00317)	Certain types of cancer	[31–33]
TC (D013964)	Erlotinib (DB00530)	Non-small cell lung cancer	[34–36]
TC (D013964)	Panitumumab (DB01269)	Antineoplastic agent	[36]
AML (D015470)	Mecasermin (DB01277)	Primary IGF 1 deficiency	n/a
TC (D013964)	Purvalanol (DB02733)	n/a	n/a

Specially, tetracycline [DrugBank:DB00759], which is the drug receiving the highest PDOD score and is not contained in our answer drug sets for oligodendroglioma, has already a supporting literature evidence for the treatment of oligodendroglioma [27]. This drug inhibits its target [Entrez ID:5621]. The distance *d*_*c*_ from its target [Entrez ID:5621] to up-regulated disease gene [Entrez ID:3280] is ‘+3’ which is obtained by two ‘activation like paths’ from DKEGG and thus its target activates up-regulated disease genes. Therefore, this drug may inhibit an up-regulated disease gene [Entrez ID:3280] of oligodendroglioma not directly but through its target [Entrez ID:5621] and DKEGG.

Five drugs, fulvestrant [DrugBank:DB00947], cetuximab [DrugBank:DB00002], gefitinib [DrugBank:DB00317], erlotinib [DrugBank:DB00530], and panitumumab [DrugBank:DB01269], do not exist in our answer drug sets but they also have a potential to treat thyroid carcinoma according to literature. All of five drugs also inhibit an up-regulated disease gene [Entrez ID:595] and activate a down-regulated disease gene [Entrez ID:83439] through their targets [Entrez ID:1956, 2099] and DKEGG. For example, fulvestrant have an antagonistic effect on its target [Entrez ID:2099]. The distance from its target [Entrez ID:2099] to up-regulated disease gene [Entrez ID:595] is ‘+1’ by one ‘activation like path’. The distance from its target [Entrez ID:2099] to down-regulated disease gene [Entrez ID:83439] is ‘-3’ by one ‘inhibition like path’. Thus, fulvestrant would inhibit an up-regulated disease genes and activate a down-regulated disease gene. For another example, gefitinib have an antagonistic effect on its target [Entrez ID:1956]. The distance from its target [Entrez ID:1956] to up-regulated disease gene [Entrez ID:595] is about ‘2.57’ by eight ‘activation like paths’ and one ‘inhibition like path’. The distance from its target [Entrez ID:2099] to down-regulated disease gene [Entrez ID:83439] is ‘-2’ by one ‘inhibition like path’. Therefore, gefitinib also would inhibit an up-regulated disease genes and activate a down-regulated disease gene and have an opposite effects on altered states of disease genes through drug-drug target interactions and paths from drug target to disease genes in DKEGG. These results not only are consistent with the previous study [26] but also indicate the importance of the consideration of biological networks in predicting indications of drugs.

### Implementation in online

For researchers to infer drugs having opposite effects on altered genes they interested, we implemented PDOD in online (http://gto.kaist.ac.kr/pdod/index.php/main). Users can submit a gene list with their Entrez gene IDs [37] and altered states which they gained from their experiments. Users can select and submit alpha values according to the disease of interest [26] or the distribution of *d*_*C*_ in Additional file 4: Figure S2. Then, users will get a drug list likely to have reverse effects on their genes if more than one gene existed in DKEGG. In addition, users can simply access our web page via mobile.

## Conclusions

In this paper, we developed a novel network-based method, PDOD, to identify novel indications of existing drugs. We inferred drugs having reverse effects on altered states of disease genes with the consideration of ‘effect type’ and ‘effect direction’ in directed network. For a given disease gene set with altered states, we predicted a drug as a medicine of the disease when the drug satisfies two conditions: 1. Target proteins of a drug are close to disease genes; 2. A drug restores altered states of disease genes through paths from a drug to disease genes. We applied our method to infer candidate therapies for nine diseases. Our PDOD is compared to two previous approaches, the network-based method without ‘effect type’, ‘effect direction’, and ‘altered states of disease gene’ information and the method which did not utilize any biological network. This comparison showed the importance of this kind of information. In addition, we represented the potentialities of some of high ranked drugs in PDOD as novel treatments of the diseases.

We constructed a web based tool implementing PDOD. Our online tool may help researchers to predict drugs having opposite effects on disease genes and generate a hypothesis easily by using PDOD. Since average values of distances between drug targets and disease genes are various according to categories of diseases [26], users can set parameters depending on the disease of interest. In addition, even though we identified altered states of disease genes using expression data, researchers can input genes with changed states from any kind of experiment.

In spite of our promising results for a case study, our works have several drawbacks. One of the major drawbacks in our approach is that the number of ‘effect types’ used in our method is limited to four interactions. Relations having ambiguous ‘effect type’ and ‘effect direction’, for example, binding, association, and phosphorylation were excluded at this present; thus, the scale of backbone network is relatively small compared to a real molecular network. We did not include these relations because they can bring out false positives in prediction of drugs; however, it causes that some diseases like multiple sclerosis cannot be applied PDOD to since genes related to these diseases do not exist in our constructed network. Another drawback of our approach is that we considered only the shortest paths although there would be longer paths from drug targets to disease genes. Even though we only used the shortest paths based on the assumption that closer genes may be more interactive, which is commonly used in other papers using biological network [38, 39], the consideration of longer paths could give us more comprehensive results.

In further study, we plan to enlarge scales of the backbone network not only by including other interactions like phosphorylation, methylation, binding/association, and metabolic reaction but also by combining other databases containing ‘effect type’ and ‘effect direction information’ such as pathway interaction database (PID) and small molecule pathway database (SMPDB). We will apply our method to more disease cases and validate promising drugs from our PDOD with wet lab experiment through collaboration. In addition, we expect that a negative score between a drug and disease in PDOD may explain the drug that is likely to have same effects on altered states of disease genes. In other words, the disease may be regarded as side effects of the drug. Based on this idea, the proposed approach may be feasible to predict side effects of drugs and drug-drug interactions.

Homeostasis is one of the most fundamental features of mankind. If a disease occurred and homeostasis could not be maintained, a drug having opposite effects on the disease could restore the altered states in patients. Our approach would be appropriate to predict these kinds of drugs.

## Additional files

Additional file 1: Figure S1. Flow diagram of PDOD. The figure that illustrates how the databases are used to generate the inputs and expected outputs. (PDF 166 kb) Additional file 2: Table S1. 16 diseases considered in this work. Statistics and information of data for 16 diseases are described in this file: Disease name; MeSH ID of diseases; GSE numbers we used; platforms of the GSE; tissues where GSE are generated; the number of disease genes from CTD; the number of differentially expressed disease genes; the number of differentially expressed disease genes in DKEGG. (XLSX 12 kb) Additional file 3: Table S2. Answer drugs used in this work for nine diseases. Information about answer drugs used in this work are described in this file: Disease name; MeSH ID of diseases; the number of answer drugs for each disease; the list of answer drugs (DrugBank ID). (XLSX 11 kb) Additional file 4: Figure S2–S3. The distribution of dc and PDOD scores. Each figure presents the distribution of dc between all genes in DKEGG and PDOD scores between all drugs and nine diseases we used. (PDF 236 kb) Additional file 5: Figure S4–S12. AUC values for nine diseases with different values of *α*. AUC values of PDD and PDOD for nine diseases according to different values of parameter *α* are enclosed in this file. (PDF 131 kb)

## Abbreviations

PDOD: Prediction of Drugs having opposite effects on Disease genes
DKEGG: Directed KEGG pathways, which we constructed, containing only directed relations such as ‘activation’ and ‘inhibition’
PDD: Prediction of Drugs having effects on Disease genes
ACL: Adenocarcinoma of lung
AML: Acute myelocytic leukemia
ACY: Astrocytoma
AS: Asthma
GB: Glioblastoma
OG: Oligodendroglioma
PD: Parkinson disease
PH: Pulmonary hypertension
SZ: Schizophrenia
TC: Thyroid carcinoma
