# Supplementary figures and images for: Prediction of drugs having opposite effects on disease genes in a directed network
Source: BMC Syst Biol. 2016 Jan 11;10(Suppl 1):2. doi: 10.1186/s12918-015-0243-2 (PMC4895308; doi:10.1186/s12918-015-0243-2)

Figure S1 – Flow diagram of PDOD

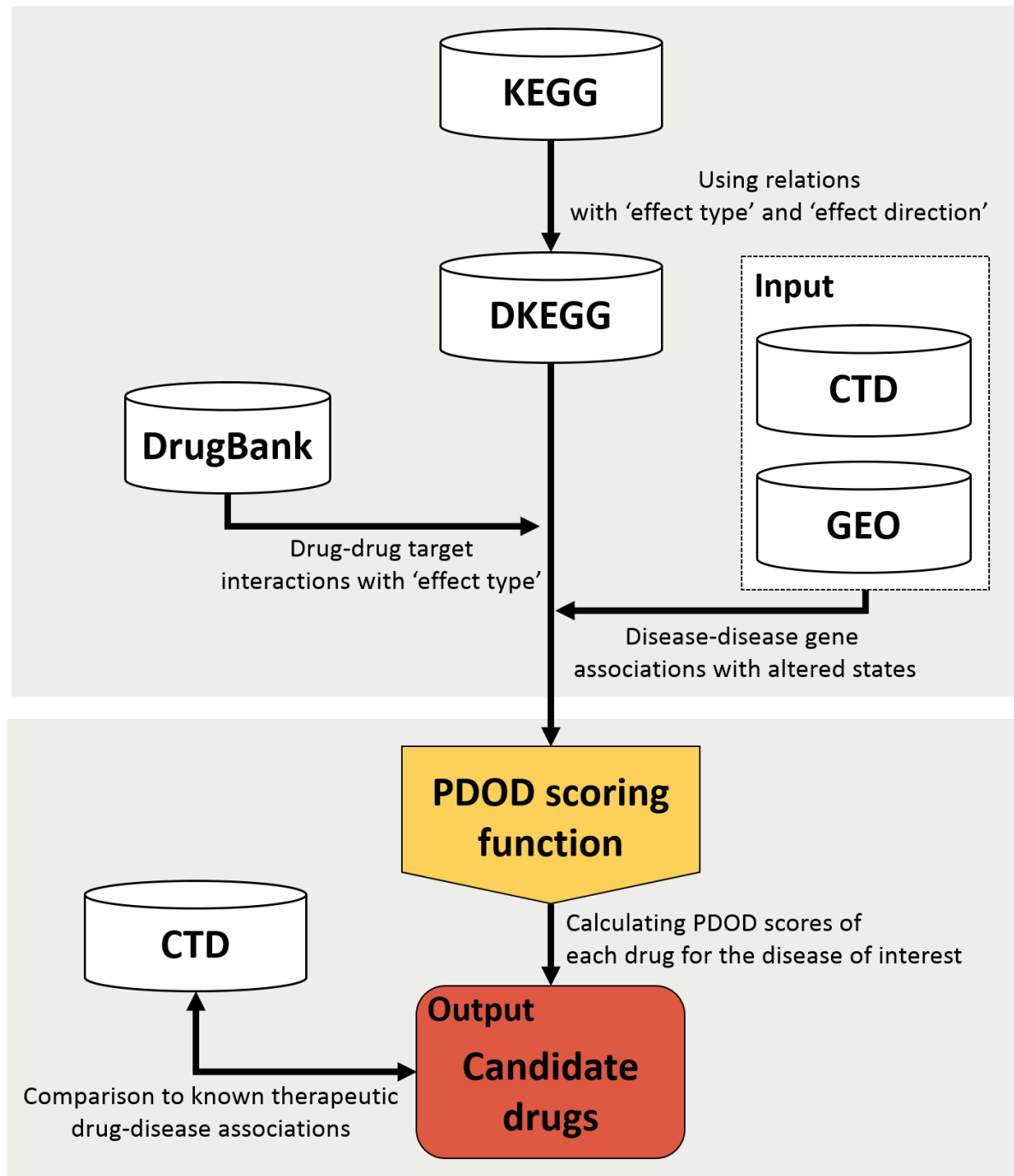

Supplement: Additional file 1: Figure S1. — Flow diagram of PDOD. The figure that illustrates how the databases are used to generate the inputs and expected outputs. (PDF 166 kb) [file 12918_2015_243_MOESM1_ESM.pdf]
