# Supplementary material for: Prediction of drugs having opposite effects on disease genes in a directed network
Source: BMC Syst Biol. 2016 Jan 11;10(Suppl 1):2. doi: 10.1186/s12918-015-0243-2 (PMC4895308; doi:10.1186/s12918-015-0243-2)
Supplement: Additional file 4: Figure S2–S3. — The distribution of dc and PDOD scores. Each figure presents the distribution of dc between all genes in DKEGG and PDOD scores between all drugs and nine diseases we used. (PDF 236 kb) [file 12918_2015_243_MOESM4_ESM.pdf]

Figure S2 – The distribution of dc between all gene pairs in DKEGG

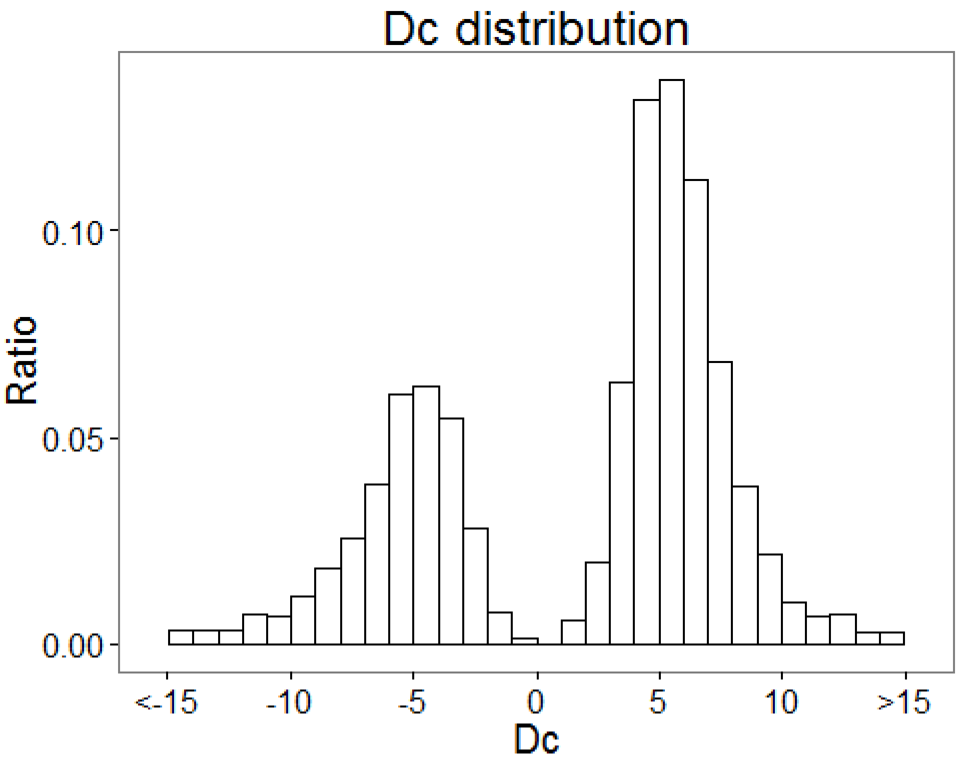

This figure shows the distribution of d_C between all gene pairs in DKEGG excluding gene pairs having an infinite distance which means that there is no path from node A to node B or the numbers of ‘activation-like paths’ and ‘inhibition-like paths’ from node A to B are same. The number of genes pairs having an infinite distance value is 7,710,274 among total 10,936,249 gene pairs. The median of d_C excluding gene pairs having infinite distance values is 4. It shows there are more ‘activation-like paths’ between genes than ‘inhibition-like paths’. The median of the absolute values of d_C excluding gene pairs having infinite distance values is 5.7.

Figure S3 – The distribution of PDOD scores between all drugs and nine diseases we used

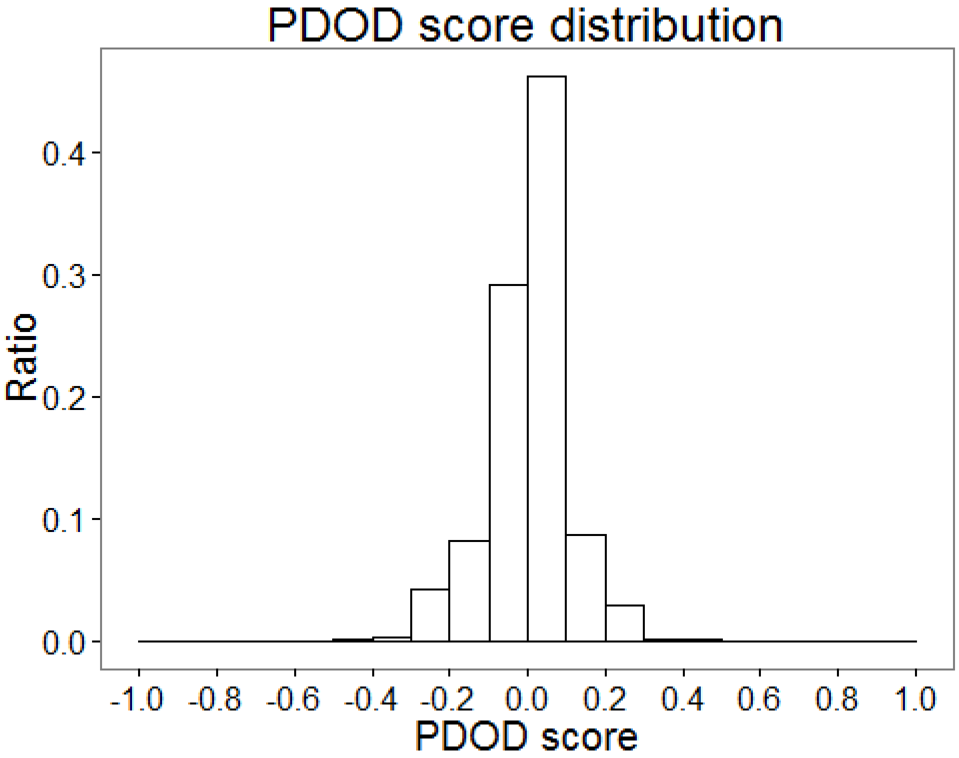

This figure shows the distribution of PDOD scores between 898 drugs and nine diseases we used, total 8082 pairs. The highest PDOD score (between Tetracycline and oligodendroglioma) is 0.5. Top 1% (the 81th drug) in PDOD scores is about 0.26 and top 5% (the 404th drug) in PDOD scores is about 0.17. The median of PDOD scores is zero.
