# Supplementary material for: Prediction of drugs having opposite effects on disease genes in a directed network
Source: BMC Syst Biol. 2016 Jan 11;10(Suppl 1):2. doi: 10.1186/s12918-015-0243-2 (PMC4895308; doi:10.1186/s12918-015-0243-2)

Figure S4 – Adenocarcinoma of lung

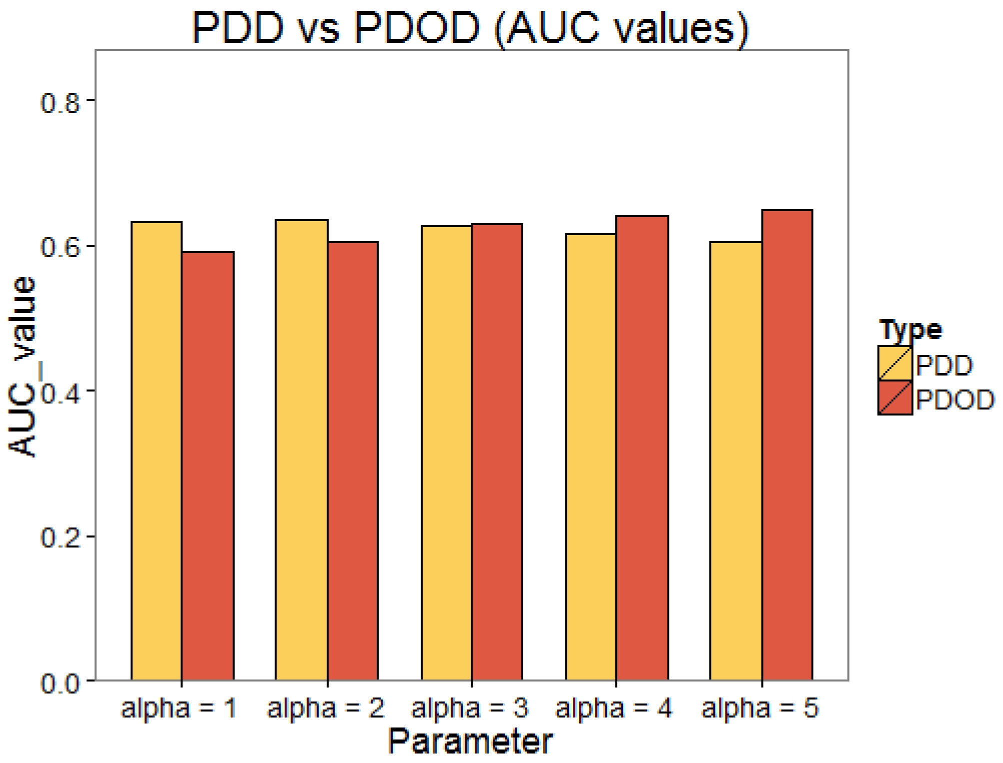

Figure S5 – Acute myelocytic leukemia

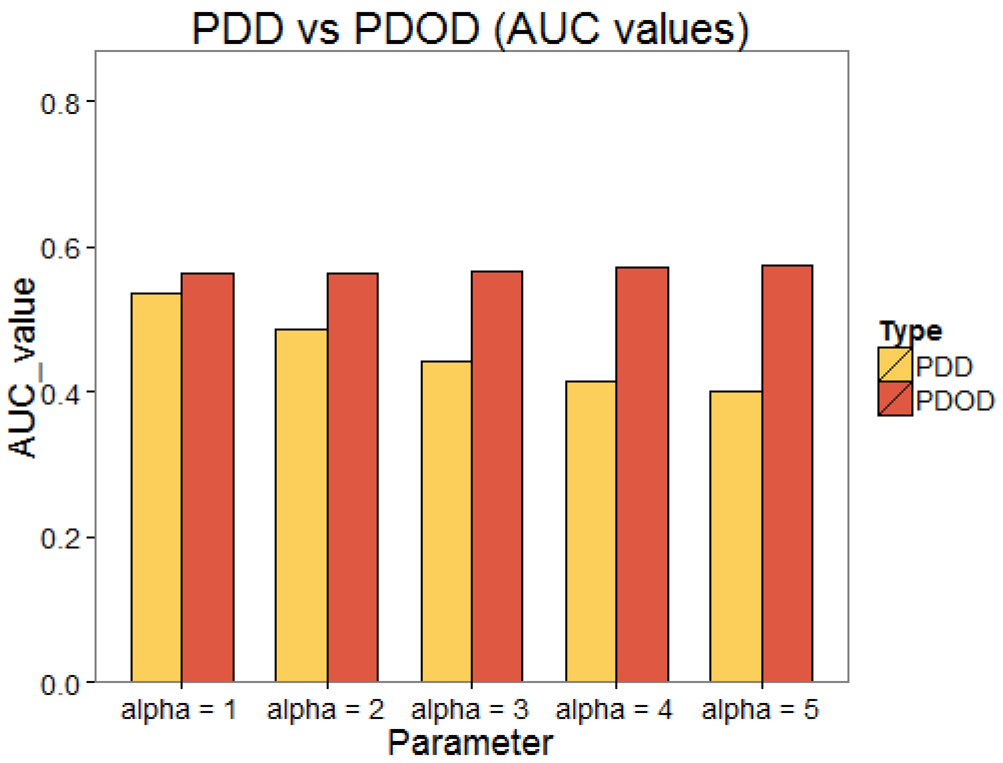

Figure S6 – Asthma

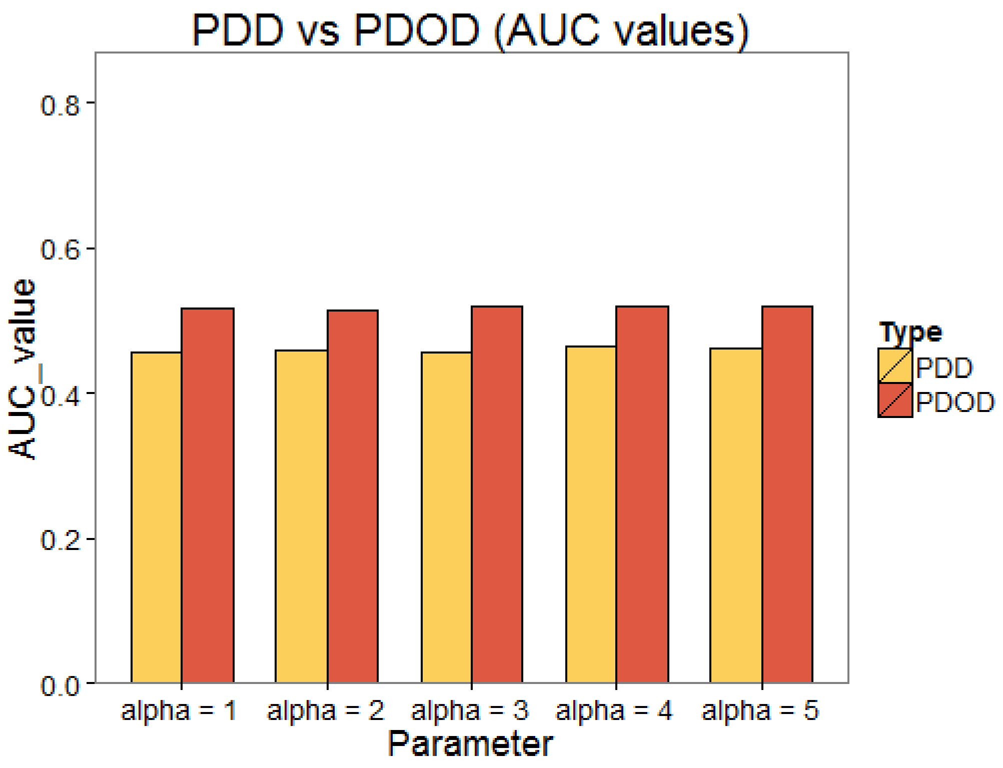

Figure S7 – Astrocytoma

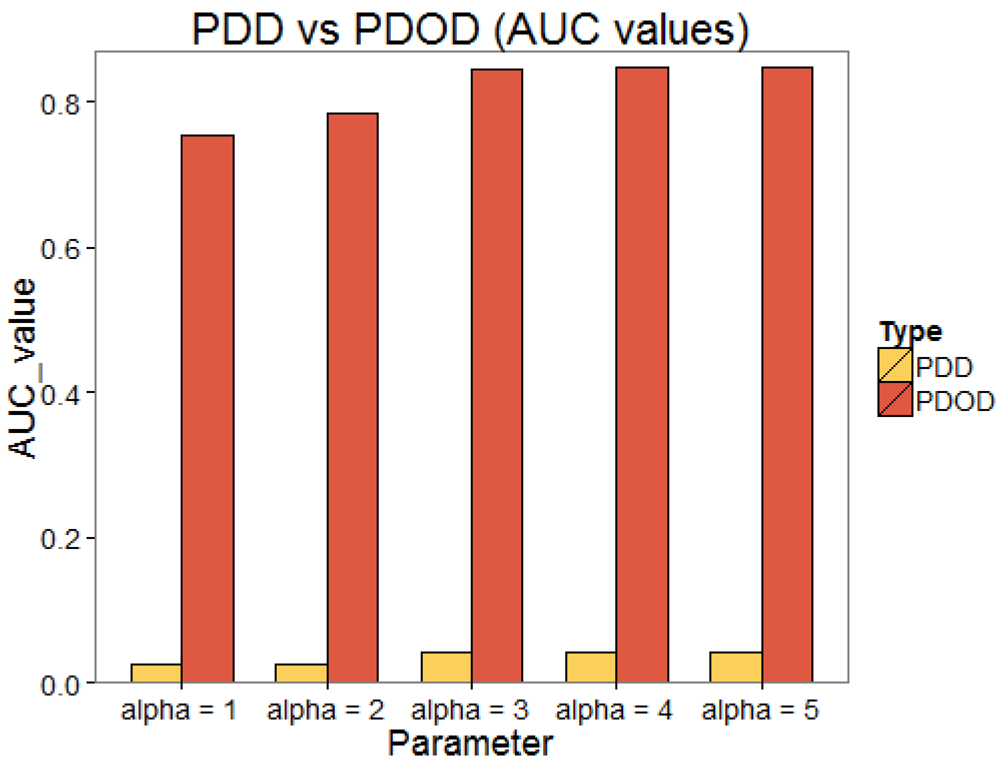

Figure S8 – Glioblastoma

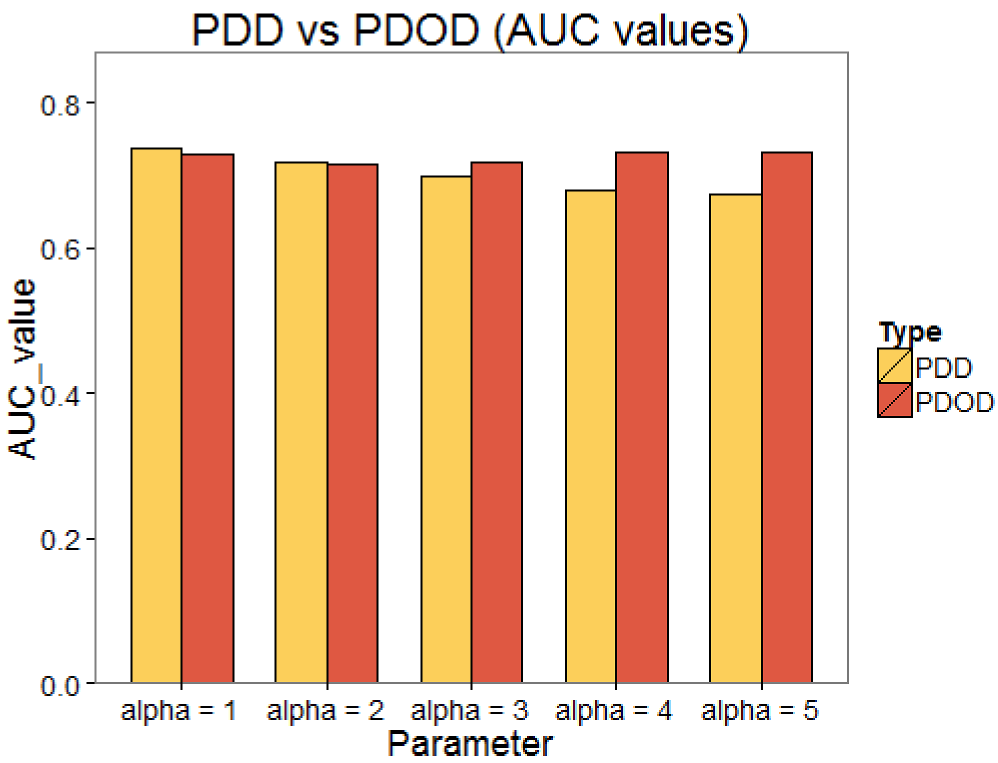

Figure S9 – Oligodendroglioma

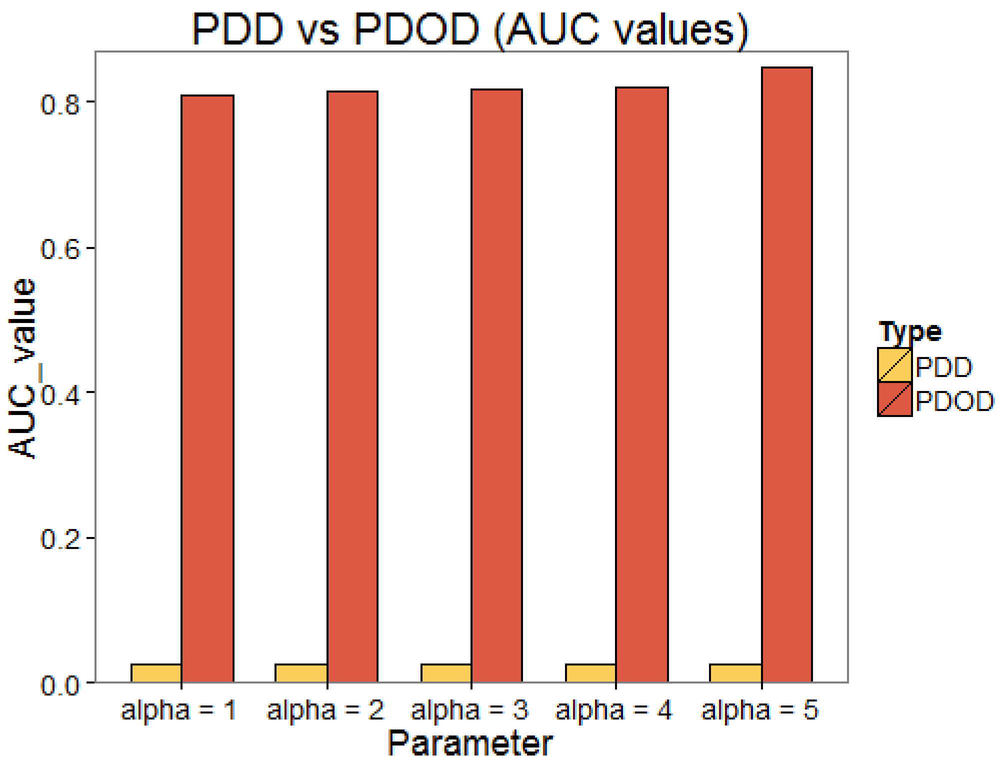

Figure S10 – Parkinson disease

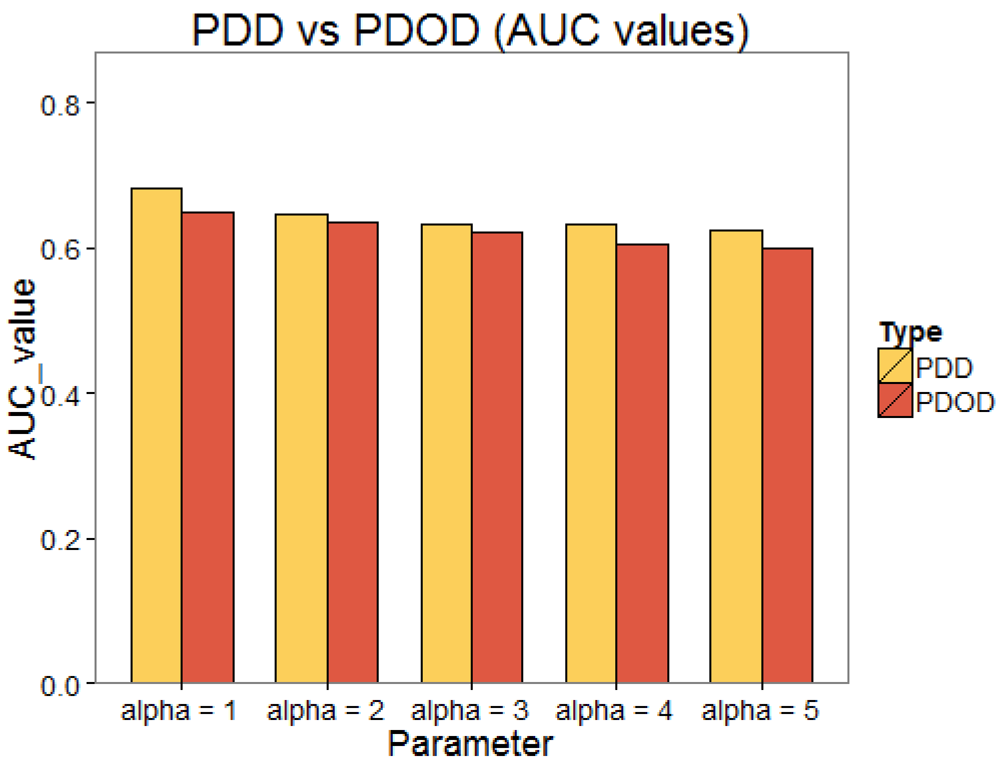

Figure S11 – Schizophrenia

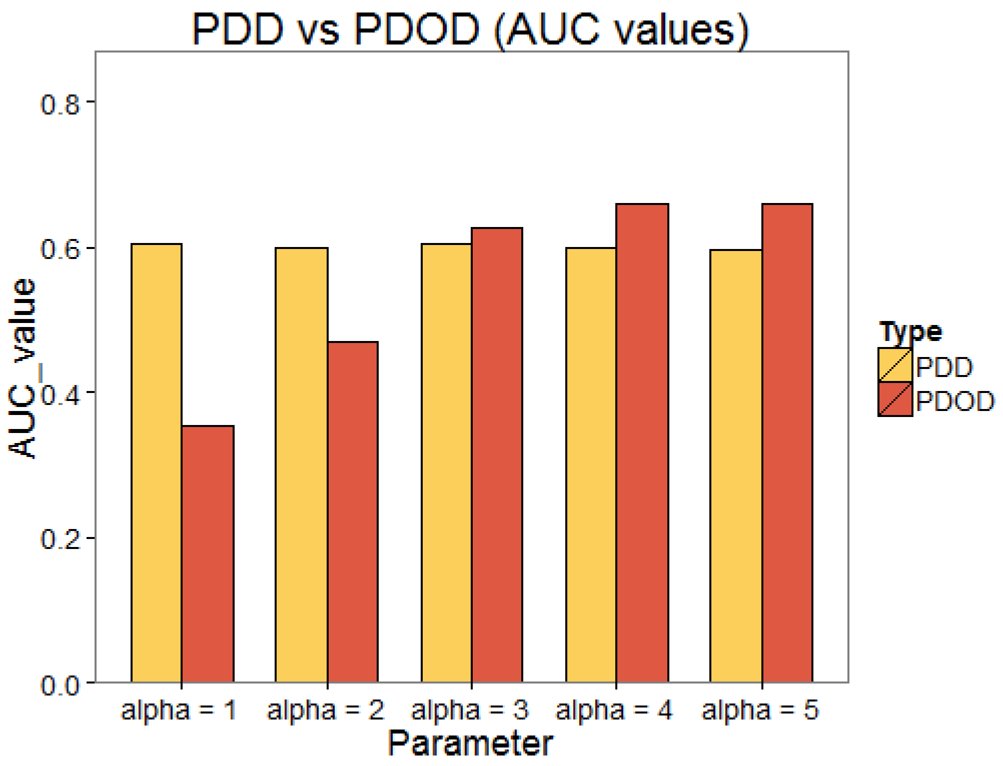

Figure S12 – Thyroid carcinoma

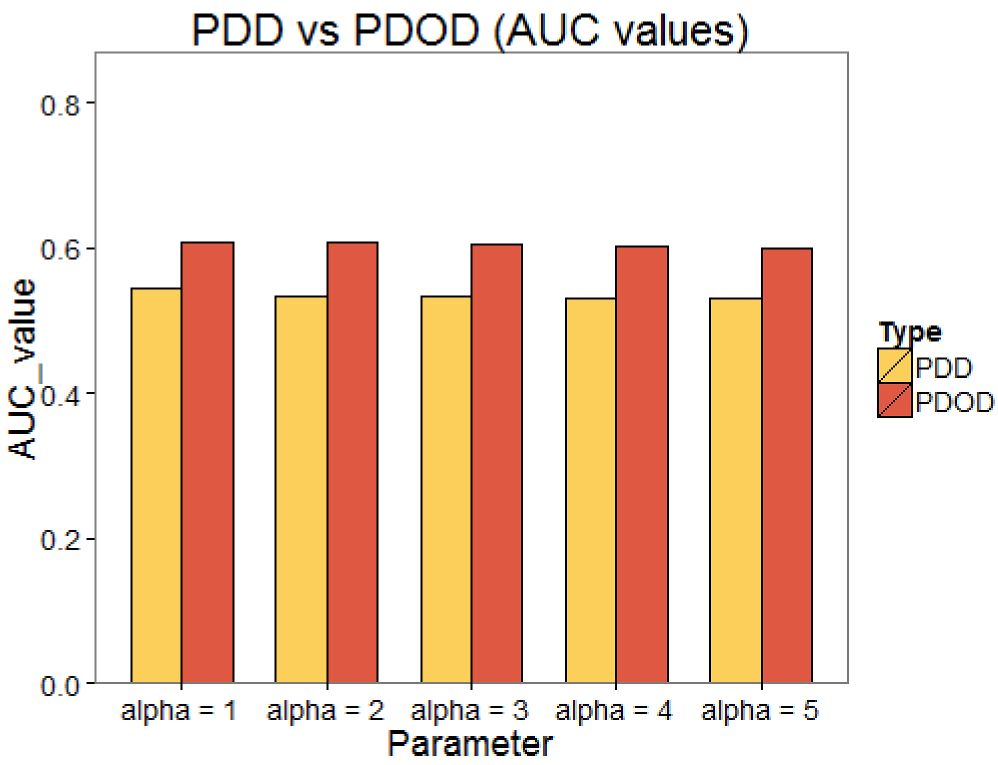

Supplement: Additional file 5: Figure S4–S12. — AUC values for nine diseases with different values of α. AUC values of PDD and PDOD for nine diseases according to different values of parameter α are enclosed in this file. (PDF 131 kb) [file 12918_2015_243_MOESM5_ESM.pdf]
